# Editorial: Artificial intelligence, biosensing, and brain stimulation in neurodegenerative diseases: progress and challenges

**DOI:** 10.3389/fnagi.2024.1362574

**Published:** 2024-01-16

**Authors:** Haiqiang Zou, Chencheng Zhang

**Affiliations:** ^1^Department of Neurosurgery, General Hospital of Southern Theatre Command, Guangzhou, China; ^2^Clinical Neuroscience Center, Ruijin Hospital Shanghai Jiaotong University School of Medicine, Shanghai, China

**Keywords:** artificial intelligence, biosensor, brain stimulation, neurodegenerative diseases, topological data analysis

## Introduction

Neurodegenerative diseases, including Alzheimer's and Parkinson's, present formidable challenges due to their progressive nature and the absence of a definitive cure. Despite available treatments to alleviate symptoms, the lack of standardized diagnostic and prognostic biomarkers complicates timely and accurate clinical interventions. The inherent inter-individual variability further complicates the diagnostic and management process for clinicians. However, recent developments in artificial intelligence (AI), biosensor systems, and innovative analysis techniques hold promise in addressing these challenges.

We launched this Research Topic aims to provide the state-of-the-art on the clinical use and role of biosensing and neuromodulation (especially AI-based) in the early detection, assessment, diagnosis, prognosis, and management of patients with neurodegenerative diseases. Due to the time limit of the Research Topic, we have selected five interesting applied clinical studies from many submitted papers.

1. Transcranial magnetic stimulation (TMS), is crucial for improving the quality of life of PD patients. However, the lack of standardized stimulation parameters makes it challenging to achieve consistent results in improving motor symptoms of PD, thereby limiting its clinical applicability. Dong et al. provide a comprehensive analysis of the effects of TMS with different frequencies and stimulation sites on motor function in PD by employing a Bayesian network meta-analysis to uncover the comparative efficacy of various TMS parameters. This review holds significant importance in optimizing the therapeutic effect of TMS in treating motor symptoms of PD. The use of Bayesian network meta-analysis in this study allows for integrating direct and indirect evidence from multiple studies, resulting in a more comprehensive analysis. This approach enables the comparison of various TMS interventions that might not have been directly compared in individual studies. Understanding the effects of TMS targeting different frequencies and stimulation sites on motor function in PD patients not only guides treatment decisions but also assists clinicians in tailoring TMS interventions based on individual patient characteristics, thereby optimizing treatment outcomes.

2. Wang et al. used three feature selection algorithms and four classification algorithms to construct an automatic model to detect PD in the early stage, which is important to clinical significance for the early diagnosis of Chinese mainland PD, based on two types of features, phonation and articulation. Although the accuracy rate is only 75.76%, this study proved that the automatic detection model is feasible and promising for Mandarin; and, articulation features are more representative than phonation features.

3. Qu et al. investigated the utility of a wearable biosensor system, the Parkinson's KinetiGraph^TM^ (PKG), in identifying and quantitatively assessing motor complications in PD among the Chinese population. The results showed that PKG assessment was significantly correlated with clinical scale assessments and displayed high sensitivity and specificity in identifying motor complications. It provided a valuable tool for managing these complications objectively, quantitatively, and remotely, which addressed the needing for effective clinical evaluation and management of PD patients with motor complications.

4. Ma, Feng et al. proposed a new approach to disease progression analysis based on topological data analysis (TDA), or the mapper algorithm to be precise. Using the mapper algorithm, we apply relatively fewer parameters to achieve better results than the previous models and provide accuracy in the 62.5–97.0% range in predicting motor progression depending on different medication types. The use of this model can predict motor evaluations at the individual level, assisting clinicians in adjusting intervention strategies for each patient and identifying at-risk patients for future disease-modifying therapy clinical trials. Our findings indicate that the models can practically predict the MDS-UPDRS Part III score of the coming year based on the clinically available characteristics obtained in the current year.

5. The Movement Disorder Society's Unified Parkinson's Disease Rating Scale Part III (MDS-UPDRS III) is most commonly used for evaluating motor symptoms of PD. Vision-based evaluation is one of the most available patterns for remote circumstances. However, two items of it, including rigidity (item 3.3) and postural stability (item 3.12) are impossible to assess in the original design of MDS-UPRS III. Ma, Shi et al. combined computer vision and machine learning to construct models for evaluating rigidity and postural stability by other motions from MDS-UPDRSIII, which can be completed by people with PD independently or with the help of their caregivers in front of the camera. This is the first study managing to assess rigidity and postural stability purely by vision and has the potential to be applied remotely.

## Concluding remarks

These studies collectively represent a significant step forward in neurodegenerative disease research, leveraging AI, biosensors, and innovative analysis techniques. Despite the challenges and imperfections, the insights provided by these studies offer potential solutions for personalized medicine in neurodegenerative diseases. The integration of technology into healthcare, as demonstrated in these articles, presents a promising avenue for improving patient outcomes and enhancing the quality of life in individuals facing neurodegenerative conditions.

## Author contributions

HZ: Writing – original draft, Writing – review & editing. CZ: Writing – review & editing.

